# Hybrid Deep-Learning and Machine-Learning Models for Predicting COVID-19

**DOI:** 10.1155/2021/9996737

**Published:** 2021-08-03

**Authors:** Talal S. Qaid, Hussein Mazaar, Mohammad Yahya H. Al-Shamri, Mohammed S. Alqahtani, Abeer A. Raweh, Wafaa Alakwaa

**Affiliations:** ^1^Computer Science Department, College of Computer Science, King Khalid University, Abha 61421, Saudi Arabia; ^2^Faculty of Computer Science and Engineering, Hodeidah University, Hodeidah, Yemen; ^3^Computer Science Department, College of Science and Arts in Tanumah, King Khalid University, Abha 61421, Saudi Arabia; ^4^Computer Engineering Department, College of Computer Science, King Khalid University, Abha 61421, Saudi Arabia; ^5^Electrical Engineering Department, Faculty of Engineering, Ibb University, Ibb, Yemen; ^6^Radiological Sciences Department, College of Applied Medical Sciences, King Khalid University, Abha 61421, Saudi Arabia; ^7^BioImaging Unit, Space Research Centre, Department of Physics and Astronomy, University of Leicester, Leicester LE1 7RH, UK

## Abstract

The COVID-19 pandemic has had a significant impact on public life and health worldwide, putting the world's healthcare systems at risk. The first step in stopping this outbreak is to detect the infection in its early stages, which will relieve the risk, control the outbreak's spread, and restore full functionality to the world's healthcare systems. Currently, PCR is the most prevalent diagnosis tool for COVID-19. However, chest X-ray images may play an essential role in detecting this disease, as they are successful for many other viral pneumonia diseases. Unfortunately, there are common features between COVID-19 and other viral pneumonia, and hence manual differentiation between them seems to be a critical problem and needs the aid of artificial intelligence. This research employs deep- and transfer-learning techniques to develop accurate, general, and robust models for detecting COVID-19. The developed models utilize either convolutional neural networks or transfer-learning models or hybridize them with powerful machine-learning techniques to exploit their full potential. For experimentation, we applied the proposed models to two data sets: the COVID-19 Radiography Database from Kaggle and a local data set from Asir Hospital, Abha, Saudi Arabia. The proposed models achieved promising results in detecting COVID-19 cases and discriminating them from normal and other viral pneumonia with excellent accuracy. The hybrid models extracted features from the flatten layer or the first hidden layer of the neural network and then fed these features into a classification algorithm. This approach enhanced the results further to full accuracy for binary COVID-19 classification and 97.8% for multiclass classification.

## 1. Introduction

COVID-19 was the most challenging health problem in 2020, following its emergence in December 2019 in Wuhan, China. Due to its global impact on populations, the World Health Organization (WHO) called it a pandemic in February 2020. By November 28, 2020, there were more than 62 million confirmed cases and 1.5 million deaths worldwide. In fact, this virus is like other coronaviruses that have appeared in the past two decades, like the middle east respiratory syndrome coronavirus (MERS-CoV) and severe acute respiratory distress syndrome coronavirus (SARS-CoV) [1, 2]. These infections are dangerous, as they spread very quickly, and hence early detection and diagnosis will hasten the response, alongside proper treatment and care. In fact, there are many other causes of viral pneumonia: lung infections like flu, the common cold, and some viruses. In its early stages, viral pneumonia sticks to the upper part of the respiratory system. The lungs' air sacs start to get infected, inflamed, and filled with fluid if the infection reaches the lungs, presenting significant health risks, especially for those with comorbidities [3, 4].

Hospital staff, doctors, nurses, and clinical facilities have many strategies and tools for diagnosing and reducing the impact of this epidemic. The most currently used technique to detect COVID-19 infection is reverse-transcription polymerase chain reaction (RT-PCR), but it has a low sensitivity of 60%–70%. Another possible diagnosis option is to use radiological images of patients using volumetric chest CT and X-ray imaging, which may help doctors analyze and predict the effects of COVID-19 on the human body. CT uses a high radiation dose, which limits its use in children and pregnant women, while X-rays use a low radiation dose at low cost. As such, the X-ray is a good candidate for imaging the lungs and may be an effective method for the early detection of COVID-19, especially in countries that cannot purchase expensive laboratory kits for COVID-19 testing [2, 5, 6]. However, discriminating between COVID-19 and other viral pneumonia is challenging because the radiographic features are similar. Moreover, the lung has complex morphological patterns that change in extent and appearance over time [4, 7, 8]. Therefore, designing artificial models to detect these patterns with high accuracy is very important to rapidly screen and see infections of COVID-19 and help radiologists by providing practical assistant tools [9]. These models use chest X-ray images of healthy lungs and those infected with COVID-19 for early detection of this disease.

For this purpose, we collected data of X-ray images of healthy and COVID-19 infected patients from different sources to test the effectiveness of the proposed models. In fact, this work focuses mainly on using convolutional neural networks (CNNs) and transfer-learning models for classifying chest X-ray images for coronavirus-infected patients. A lack of availability of many pictures of COVID-19 patients has made detailed studies about solutions for automatic detection of COVID-19 from X-ray (or chest CT) images rare. Moreover, labeling such images for deep-learning (DL) applications is not an easy job and seems expensive [10, 11]. Small data sets of COVID-19 X-ray images have been announced for AI researchers to train machine-learning (ML) models to perform automatic COVID-19 diagnoses from X-ray images [12]. Recently, Sedik et al. [10] collected a data set of 6,128 X-ray, CT, and ultrasonic lung images, but they were mixed and imbalanced between training and testing. The data sets available are still small, and hence two enhancement strategies were adopted in this paper to address the scarcity of COVID-19 X-ray images:

1. We used data augmentation to create transformed versions of COVID-19 X-ray images (such as flipping, slight rotation, and adding a small amount of distortion) to increase the number of samples by a factor of 5.
2. Instead of training our models from scratch, we fine-tuned the last layer of the pretrained version of these models on ImageNet. This way, it was possible to train the model with fewer labeled samples from each class.

The paper is organized as follows: the literature review of previous work on COVID-19 is presented in Section 2. The data set used in this paper and its characteristics are described in Section 3. The proposed models' architectures are defined and discussed in detail in Section 4. This section presents four different models that show promising results for both binary and multiclass classifications. Simulations and discussions are summarized in Section 5, and finally, the paper is concluded in the last section.

## 2. Literature Review

Research on medical image-processing using DL started in 1995, classifying lung nodules using X-ray images [13]. Apostolopoulos and Mpesiana [14] collected X-ray images from North America and Italy and fed them into a CNN model for COVID-19 detection. Ozturk et al. [5] proposed the DarkNet model for detecting COVID-19 cases via viral pneumonia images and identifying a location if there is a shortage of radiologists due to the enormous number of patients. Rajpurkar et al. [15] presented a CheXNet model to diagnose lung diseases using DL for processing X-ray images. Karakanis and Leontidis [16] built a network to augment X-ray data with synthetic images and proposed DL models for binary and multiclass classifications of COVID-19. Another work in such a direction was done by Das et al. [17] using DL and transfer-learning (TL) for building models to be tested on Kaggle data sets. Hussain et al. [18] proposed a TLCoV model to detect COVID-19 using CT scan and X-ray images automatically and called it CoroDet. They argued that their COVID-19 classification model was accurate for both binary and multiclass classifications.

EMCNet was proposed by [19] to detect COVID-19 cases by evaluating chest X-ray images. They used a CNN for extracting deep features of the photos and then used binary-classification techniques. They combined the outputs of many classifiers to form an ensemble for better detection capabilities. Sedik et al. [10] used a CNN and convolutional long short-term memory for building a DL model to detect COVID-19 cases. They tested their models on two data sets—X-rays and CT scans—with normal, COVID-19, and pneumonia classes. They added some ultrasound images to their data set and argued that their models could be used for quick detection of COVID-19. Maior et al. [11] discussed the effect of limited X-ray images for COVID-19 detection. They tried to resolve this problem by combining different data sets and used them for testing CNN models. Sedik et al. [20] proposed two models for augmenting images to increase the learning ability of some DL methodologies, which enhance the detection possibility of COVID-19 cases. Many other researchers have investigated many techniques based on ML and DL to detect COVID-19 based on X-ray and CT images for public data sets, as listed in Table 1.

## 3. Data Sets

As we discussed before, collecting X-ray images for COVID-19 is still in its early stages. For enhancing the number of samples for our experiments, we merged two real data sets. The first dataset was collected from Kaggle. We downloaded the database of chest X-ray images for positive cases of COVID-19, along with viral pneumonia and normal images. There were 219 COVID-19 positive images, 1,345 viral pneumonia images, and 1,341 normal images for this data set. Downloading viral pneumonia images allowed us to test our model in differentiating COVID-19 from other viral pneumonia infections, as it is possible to guide the system to the wrong decision. The second data set, on COVID-19 patients, was collected from Asir Hospital in Saudi Arabia.

**Figure 1:**
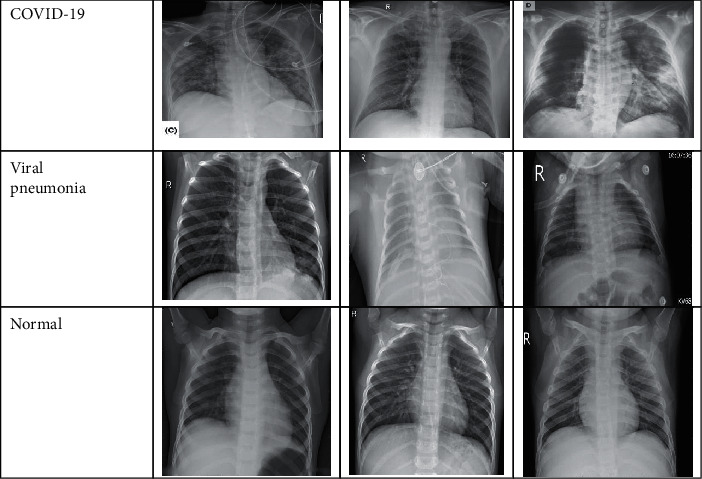
X-ray samples of COVID-19 data set.

We augmented images annotated positive in the Kaggle data set to generate more general and robust models due to the limited positive cases. We applied augmentation techniques on the assembled data set, such as random rotation and vertical flip operations, using the ImageDataGenerator function of the TensorFlow Keras framework. We generated 657 cases and combined all images together to get a final data set of 4,103 X-ray images. Each image in the data set was resized to 120 × 120 pixels to reduce space and computation time and hence derive consistent data. Additionally, image normalization was applied to scale pixel intensities to a range of 0–255.  Table 2 describes the counts of X-ray images of each class and the distribution of each data set versus the total number of images. Some samples of the data set for different classes are shown in Figure 1.

## 4. Model Architectures

As an advanced technology, DL tries to simulate the way the human brain's neurons work. DL consists of deep convolutional and deep neural network layers. In fact, CNNs are preferred for image-processing applications, as they are robust context learners and usually extract powerful features from the data [21, 22]. Apart from its outstanding accuracy, DL requires considerable computation, memory, and time to train the model, as it has many layers and thousands, if not millions, of weights to be learned. Therefore, TL can be used to shorten such restrictions and enhance accuracy. The following sections discuss different models for detecting COVID-19 infection through X-ray images using CNNs or TL. We need our models to classify the X-ray images into normal, pneumonia, or COVID-19. We used three basic blocks for building variants of our models:

1. CNN block
2. TL block using VGG16 or VGG19
3. ML block

In the following subsections, we discuss each model in detail and illustrate its main blocks.

### 4.1. Model 1: Convolutional Neural Networks

**Figure 2:**
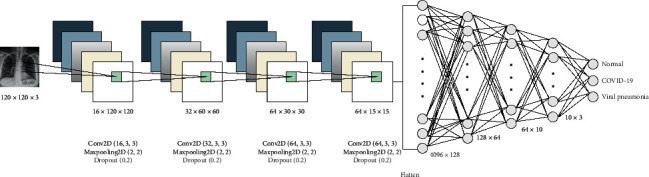
CNN model (Model 1) architecture and configuration.

DL has proven to be a highly accurate technique to guarantee high-level detection and prediction of many medical cases by extracting deep features from the data set on hand [28, 29]. We built a CNN model and trained it many times with different parameters to select the best hyperparameters. The final model consisted of four convolutional layers and four dense layers. The convolutional layers were one layer with 16 filters, one layer with 32 filters, and two layers with 64 filters. All filters were of size 3 × 3, and all convolutional layers followed maximum pooling of 2 × 2. The four dense layers were three hidden layers with 128, 64, and 10 neurons and one output layer. Details of these configurations are given in Figure 2. This model has three classification processes: binary classification between COVID-19 and normal cases, binary classification between COVID-19 and viral pneumonia cases, and multiclass classification among normal, viral pneumonia, and COVID-19 cases. As such, it will be possible to investigate the ability of our model to differentiate COVID-19 infection from normal and other viral pneumonia infections.

### 4.2. Model 2: Transfer Learning Using VGG16 or VGG19

A CNN needs vast computing and time resources to train a robust model. TL gives a shortcut for many tasks to reduce these computing and time requirements by relying on a pretrained model of other similar jobs [30]. In fact, TL models have already been trained for days or weeks and hence are perfect candidates as starting points for many tasks.

In this paper, we used VGG16 or VGG19 as TL models for building our model. However, our aim here was to enhance the system's accuracy, not to reduce training time, and thus we operated all models on the same data set without any reductions.

**Figure 3:**
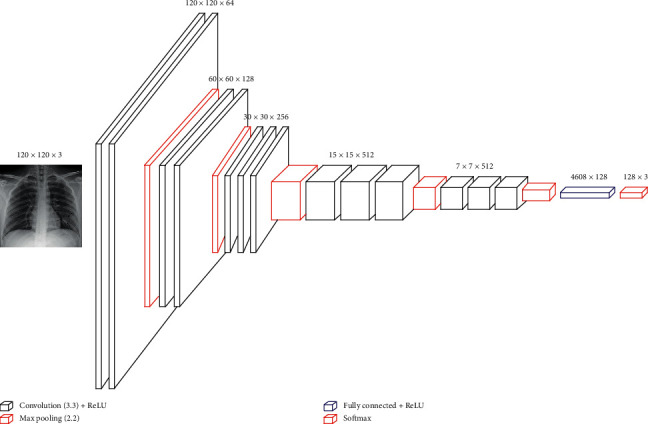
Transfer-learning models (Model 2a and Model 2b) architecture and configuration.

Initially, VGG16 was developed for the recognition of large-scale images. It used the ImageNet data set to overcome training time and insufficient data. Hussain et al. [18] showed that VGG16 outperformed other approaches they had tested. VGG16 is a CNN architecture that has 16 layers, 13 convolutional layers, and three dense layers. Our model kept all the convolutional layers with their parameters and reduced the dense layers to two only. The weights of the dense layers were trained using the data set. The convolutional layers of VGG16 were divided into five convolutional phases: two layers with 64 filters, two layers with 128 filters, three layers with 256 filters, three layers with 512 filters. All filters were of size 3 × 3, and each convolutional phase followed maximum pooling of 2 × 2. The two dense layers were one hidden layer with 128 neurons and one output layer. VGG16 has approximately 138 million parameters for the network. The details are shown in Figure 3.

Another TL model, VGG19, can be used instead of VGG16. VGG19 is a 19-layer model that adds one extra layer to each phase of the last three convolutional phases of VGG16. We followed the same strategy of using VGG16 for our models by maintaining the convolutional layers and reducing the dense layers to only two.

### 4.3. Model 3: Hybrid CNN with Machine Learning

**Figure 4:**
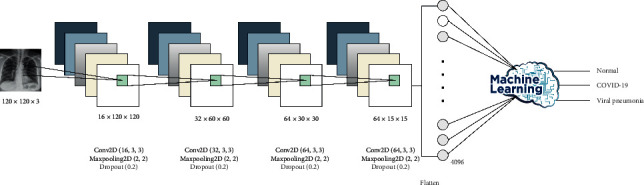
Hybrid model CNN with ML (Model 3a) architecture and configuration after the flatten layer.

**Figure 5:**
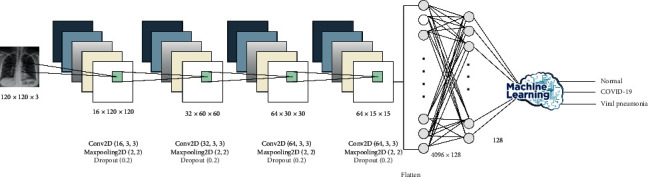
Hybrid model CNN with ML (Model 3b) architecture and configuration after the first hidden layer.

Since ML techniques need extracted features to complete the classification tasks, we decided to obtain these features from a robust technique like DL. This model extracted features using a CNN and then fed these features to one of the ML techniques. It hybridized two blocks—CNN and ML—with one used as a feature-extraction block and the other for the classification process. We examined four supervised classification techniques (naïve Bayes, support vector machine, random forest, and XGBoost). In sum, 4,096 features were extracted after the flatten layer (Model 3a), as shown in Figure 4, or 128 features after the first hidden layer of the neural network (Model 3b), as shown in Figure 5. These extracted features were used as inputs for ML. Basically, this method was used for obtaining a solid learner with the help of convolutional layers to extract features and ML to classify the results.

### 4.4. Model 4: Hybrid VGG16 with ML

**Figure 6:**
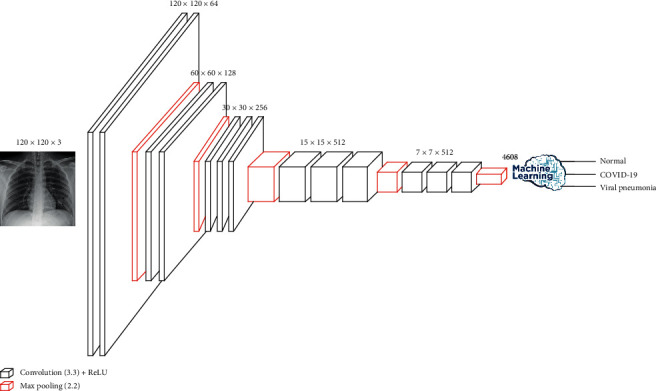
Hybrid model VGG16 with ML (Model 4a and Model 4b) architecture and configuration.

This model extracted features using a pretrained model (VGG16) because it had performed better than VGG19 for model 2. Features were extracted after the flatten layer (4,608 features, Model 4a) or after the first hidden layer of the neural network (128 features, Model 4b). Then, the features extracted were used as inputs for ML. Like Model 2, we kept all the convolutional layers with their pretrained parameters and fine-tuned the parameters of the dense layer. More details are shown in Figure 6.

## 5. Simulation and Computational Experiments

This section discusses in detail data and experiment preparation, evaluation metrics, and finally, the results and discussion.  Table 3 lists the complete set of the baseline and proposed models, along with descriptions.

### 5.1. Data and Experiment Preparation

The experiments were applied on one open-source COVID-19 data set, as described in Section 3, and local COVID-19 patients' X-ray images from Asir Hospital, Abha, Saudi Arabia. The total number of pictures of COVID-19 was 219 after being increasing by augmentation. The images generated from augmentation numbered 643. We split the data into training data (3,279) images to build the model and validation data (820 images: 304 COVID-19, 270 normal, and 246 other viral pneumonia) to tune, monitor, and select the best parameters of the model. We fine-tuned each model for 20 epochs, and the batch size was set to 32. We used a categorical/binary cross-entropy loss function and ADAM optimizer to optimize the learning function with a learning rate of 0.001. We used the regularizers *l*1 and *l*2 (*l*1 = 1*e* − 5, *l*2 = 1*e* − 4) in dense layers and dropout (0.2) after convolutional layers to avoid overfitting during the model's training. All images were downsampled to 120 × 120 before being fed to the models. Overfitting is a general problem in DL and occurs when the model fits too well to the training set because of the increasing number of features compared to the small number of samples. In this study, two approaches were used to solve this problem:

1. Dropout regularization is used for reducing overfitting and improving the generalization of deep neural networks. The network becomes less sensitive to the specific weights of neurons, becomes more capable of better generalization, and is less likely to overfit the training data. In our experiments, dropout parameters were set to 0.2.
2. Regularization in dense layers: regularizers allow us to apply penalties on layer parameters or layer activity during optimization. These penalties are summed into the loss function that the network optimizes. In our implementation, the *L*1 regularization penalty was set to 0.0001 and *L*2 to 0.00001.

### 5.2. Evaluation Metrics

The experiments for our models were evaluated using accuracy, precision, recall, and *F*1 score [31]:

(1) $$\begin{array}{c} \text{accuracy}=\frac{\text{TP}+\text{TN}}{\text{TP}+\text{TN}+\text{FP}+\text{FN}}, \end{array}$$

where TP is true positive (the number of correctly classified images of a class), TN true negative (the number of images that did not belong to a class and were not classified as belonging to that class), FP false positive (the number of wrongly classified images of a class), and FN false negative (the number of images of a class detected as another class). Precision and recall were defined; thus,

(2) $$\begin{array}{c} \text{precision}=\frac{\text{TP}}{\text{TP}+\text{FP}},\\ \text{recall}=\frac{\text{TP}}{\text{TP}+\text{FN}}. \end{array}$$

*F*1 score is defined as

(3) $$\begin{array}{c} F1\text{ score}=\frac{2\times \text{precision}\times \text{recall}}{\text{precision}+\text{recall}}. \end{array}$$

### 5.3. Experiments Conducted

We conducted extensive experiments to verify the suitability of our models, as illustrated in Table 4.

As a baseline, we implemented ConvNet#4, as it is the best model of Sekeroglu and Ozsahin [6]. This model, Model 1, Model 2a, and Model 2b were implemented three times, one to differentiate COVID-19 from normal cases, one to differentiate COVID-19 from other viral pneumonia cases, and one to differentiate COVID-19 from normal and other viral pneumonia cases. Hybrid models (3a, 3b, 4a, and 4b) were implemented once for each classifier. The total number of experiments was 44, covering all models, different approaches, and different classifiers.

## 6. Results and Discussion

**Figure 7:**
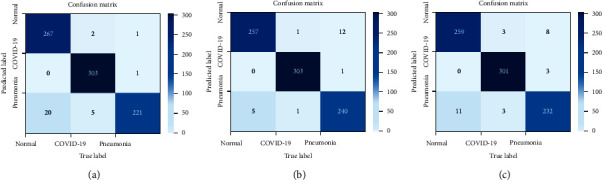
Confusion matrix of different models. (a) Model 1 (CNN). (b) Model 2a (VGG16). (c) Model 2b (VGG19).

Table 5 shows the results of the different evaluation metrics for the baseline CNN model, Model 1 (CNN), and Model 2 (VGG16 and VGG19). The baseline model's accuracy for binary classifications was high (>98%), while it was lower for multiclass classification (around 93%). Multiclass classification accuracy was 96.1%, 97.6%, and 96.6% for the proposed CNNs, VGG16, and VGG19 models, respectively. These values were higher for the other two binary-classification scenarios. Performance in terms of precision, recall, and *F*1 score was very good, with the lowest value of 95.8%. This indicated that Models 1 and 2 are efficient in detecting COVID-19 cases compared to either normal or other viral pneumonia cases. These results were better than that of the baseline model by a good margin. The confusion-matrix plots for Models 1, 2a, and 2b are depicted in Figure 7. The rows correspond to the predicted class (output class), and the columns correspond to the proper class (target class). The diagonal cells in the confusion matrix correspond to correctly classified observations (TP and TN). The off-diagonal cells correspond to incorrectly classified observations (FP and FN). The number of observations is shown inside each cell. From these results, the misclassification rate was very low for all models.

**Figure 8:**
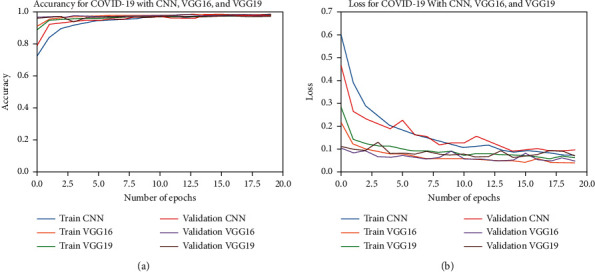
Accuracy and loss results of training and validation learning against the number of epochs. (a) Accuracy. (b) Loss.

To further study the overfitting behavior of our models, we depict the accuracy and loss results for training and validation learning for each epoch in Figure 8. The figures showed no overfitting in the models' performance due to the slight differences between the accuracy and loss of training and validation sets.

The results of the hybrid models between CNN and ML are listed in Table 6 for binary classification and in Table 7 for multiclass classification. We implemented the model four times for both scenarios, one for each ML technique, by taking the features from the flatten layer and four times by taking the features from the first hidden layer. The results showed accurate model performance, with overall binary accuracy of 100% for Model 3a with SVM. Model 3b showed 100% accuracy with SVM, naive Bayes, and random forest binary classifiers and 99% with the XGBoost binary classifier. Similarly, accuracy was very good for multiclass classifiers compared to baseline and previous models, especially Model 3b, which had only 128 extracted features from the first hidden layer of the neural network. The accuracy of Model 3a for many binary classifiers was 100%.

The results of the hybrid models between VGG16 and ML are listed in Table 8 for binary classification and Table 9 for multiclass classification. For both scenarios and each classifier, Model 4a was implemented by taking the features from the flatten layer, and Model 4b is by taking them from the first hidden layer. The results showed accurate model performance, with an overall accuracy of 100% for Model 4a with SVM and random forest binary classifiers, while Model 4b shows 100% accuracy with all examined binary classifiers. However, the performance of Models 4a and 4b was less than that of Models 2a, 3a, and 3b with multiclass classifiers.

To test the generality of the proposed models, we implemented them on another binary-class data set called “combined COVID-19 data set” [32]. This data set is a mix of X-ray, CT, and ultrasound images. This data set was augmented to generate 6,128 images and was divided into training and validation data sets as per Table 10.

Table 11 lists the accuracy values of the proposed models on the combined COVID-19 data set. The proposed models performed very well on this mixed data set, with Model 4b showing the best result with 99.6% accuracy. This proves the generality of our models and their effectiveness for the correct classification of COVID-19 cases. The previous results show that the proposed models are general, robust, and accurate for many medical images, revealing excellent and promising results. These models were built based on current technology and were tested on different general data sets.

## 7. Conclusions and Future Work

This paper presents various models for early diagnosis and classification of COVID-19 patients based on X-ray images. The models were built using CNNs, TL with VGG16 and VGG19, and ML techniques. We identified the best hyperparameters for the proposed models and exploited the power of DL to extract deep features for binary and multiclass classifiers to improve COVID-19 diagnosis accuracy. For correct prediction, we tested our models on a data set with three classes to ensure that the models were accurate in differentiating COVID-19 from other viral pneumonia infections, which have many common radiographic and ambiguous features. The proposed models outperformed the baseline one and showed promising results, especially hybrid models, which revealed very good results for both types of classifiers. For binary classification, they offered full accuracy for many cases. This illustrates the ability of DL techniques to extract relevant features, which makes the job of ML easier.

In future work, we plan to explore more X-ray data sets from different countries. Other medical images like CT scans and ultrasound images can be used for improving the accuracy of the diagnosis process and early detection models, which in turn can empower the decision-making process regarding COVID-19 patients. Moreover, other TL models can be applied with different configurations to get better results.

## Figures and Tables

**Table 1:** Literature review summary.

Author	Data set size	Image type	Disease type	ML	CNN model	TL models	Date	Max. accuracy
Minaee et al. [21]	5000	X-ray and CT	COVID-19	No	No	ResNet50, ResNet18, DenseNet-121, and SqueezeNet	2020	98%
Jain et al. [22]	6432	X-ray and CT	COVID-19	No	No	Xception	2020	97.97%
Hussain et al. [23]	558	X-ray	COVID-19 and viral pneumonia	Yes	No	No	2020	97.56%
Sekeroglu et al. [6]	6200	X-ray	COVID-19 and viral pneumonia	Yes	Yes	VGG19, MobileNet, inception, Xception, and inception ResNet	2020	99.18%
Linda Wang et al. [24]	13,975	X-ray	COVID-19 and viral pneumonia	No	Yes	VGG-19 and ResNet-50	2020	98.9%
Dingding Wang et al. [25]	1102	X-ray	COVID-19	Yes	No	VGG-16, Xception, ResNet50, and DenseNet121	2020	99.38%
Rahimzadeh et al. [26]	11302	X-ray	COVID-19 and viral pneumonia	No	No	Xception and ResNet50V2	2020	99.50%
Rahul et al. [27]	5840	X-ray	COVID-19 and viral pneumonia	Yes	No	ResNet152	2020	97.7%
Our work	4646	X-ray	COVID-19 and viral pneumonia	Yes	Yes	VGG16, VGG19, ReNet50, and MobileNet	2021	99.82

**Table 2:** Data set of this paper.

Distribution	COVID-19	Viral pneumonia	Normal	Total
COVID-19 Radiography Database (Kaggle)	219	1345	1341	2905
Asir Hospital (Saudi Arabia)	541	--	--	541
Augmented	657	--	--	657
Total	**1417**	**1345**	**1341**	**4103**

**Table 3:** Types and descriptions of the examined models.

Model	Description
Baseline	ConvNet#4: the best model proposed by Sekeroglu and Ozsahin in [6]
Model 1	The proposed CNN model
Model 2a	The proposed TL model with VGG16
Model 2b	The proposed TL model with VGG19
Model 3a	The proposed hybrid model of CNN with ML where the features are extracted from the flatten layer
Model 3b	The proposed hybrid model of CNN with ML where the features are extracted from the first hidden layer
Model 4a	The proposed hybrid model of VGG16 with ML where the features are extracted from the flatten layer
Model 4b	The proposed hybrid model of VGG16 with ML where the features are extracted from the first hidden layer

**Table 4:** Number of experiments implemented for each model.

Model	Experiments		
	Binary	Multiclass	Total
Baseline	2	1	3
Model 1	2	1	3
Model 2a	2 (VGG16)	1 (VGG16)	3
Model 2b	2 (VGG19)	1 (VGG19)	3
Model 3a	4	4	8
Model 3b	4	4	8
Model 4a	4	4	8
Model 4b	4	4	8
Total			44

**Table 5:** Results of baseline, Model 1, Model 2a, and Model 2b.

Classes	Accuracy	Precision	Recall	*F*1 score
*Baseline*				
COVID-19 vs. normal	98.82	98.87	98.77	98.82
COVID-19 vs. pneumonia	97.25	97.20	97.29	97.24
COVID-19 vs. normal vs. pneumonia	93.95	94.09	93.85	93.91

*CNN*				
COVID-19 vs. normal	99.27	98.62	100	99.30
COVID-19 vs. pneumonia	98.73	99.29	98.25	98.77
COVID-19 vs. normal vs. pneumonia	96.10	96.20	95.80	95.90

*VGG16*				
COVID-19 vs. normal	99.82	99.65	100	99.82
COVID-19 vs. pneumonia	99.45	99.65	99.30	99.47
COVID-19 vs. normal vs. pneumonia	97.60	97.40	97.50	97.40

*VGG19*				
COVID-19 vs. normal	100	100	100	100
COVID-19 vs. pneumonia	99.45	99.65	99.30	99.47
COVID-19 vs. normal vs. pneumonia	96.60	96.50	96.40	96.40

**Table 6:** Binary-classification results of Model 3a and Model 3b for different evaluation metrics.

	Classifier	Accuracy	Precision	Recall	*F*1 score
Model 3a	Naïve Bayes	94.0	94.0	94.0	94.0
	SVM	100	100	100	100
	Random forest	99.0	99.0	99.0	99.0
	XGBoost	99.0	99.0	99.0	99.0

Model 3b	Naive Bayes	100	100	100	100
	SVM	100	100	100	100
	Random forest	100	100	100	100
	XGBoost	99	99	99	99

**Table 7:** Multiclass classification results of Model 3a and Model 3b for different evaluation metrics.

	Classifier	Accuracy	Precision	Recall	*F*1 score
Model 3a	Naïve Bayes	89.8	89.7	89.3	89.5
	SVM	97.9	97.9	97.8	97.8
	Random forest	88.8	88.5	88.5	88.4
	XGBoost	94.6	94.5	94.4	94.4

Model 3b	Naïve Bayes	97.3	97.3	97.2	97.2
	SVM	97.6	97.5	97.5	97.5
	Random forest	97.8	97.8	97.7	97.7
	XGBoost	97.6	97.5	97.5	97.5

**Table 8:** Binary-classification results of Model 4a and Model 4b for different evaluation metrics.

	Classifier	Accuracy	Precision	Recall	*F*1 score
Model 3a	Naïve Bayes	95	95	95	95
	SVM	100	100	100	100
	Random forest	100	100	100	100
	XGBoost	99	99	99	99

Model 3b	Naïve Bayes	100	100	100	100
	SVM	100	100	100	100
	Random forest	100	100	100	100
	XGBoost	100	100	100	100

**Table 9:** Multiclass classification results of Model 4a and Model 4b for different evaluation metrics.

	Classifier	Accuracy	Precision	Recall	*F*1 score
Model 3a	Naïve Bayes	80.5	80.7	79.8	79.3
	SVM	96.0	95.8	95.8	95.8
	Random forest	92.4	92.3	92.2	92.1
	XGBoost	94.3	94.1	94.0	94.0

Model 3b	Naïve Bayes	96.0	95.8	95.8	95.8
	SVM	96.8	96.7	96.6	96.7
	Random forest	96.7	96.6	96.6	96.6
	XGBoost	96.7	96.5	96.6	96.6

**Table 10:** Mendeley data set: combined COVID-19 data set.

Data set	Normal class	COVID-19 class	Total
Train	2486	1711	4197
Test	1141	790	1931
Total	3627	2501	6128

**Table 11:** Accuracy results on the combined COVID-19 data set.

Model type	Accuracy
Model 1	98.7
Model 2a	98.6
Model 3b	98.5
Model 4b	99.6

## Data Availability

The data set used in this research is available on the Mendeley Data website under the name Covid-19.zip.
